# Use of Microsoft Power BI to display pregnancy related performance statistics within NHS trusts

**DOI:** 10.23889/ijpds.v8i2.2342

**Published:** 2023-09-14

**Authors:** Oliver Hugh, Jason Gardosi

**Affiliations:** 1 Perinatal Institute, Birmingham, United Kingdom

## Objectives

We wanted to create a dashboard that allowed midwives and doctors to monitor the performance of their NHS Trust by viewing the statistics relating to their performance in identifying babies at risk due to not growing well in the womb (small for gestational age, SGA), the single largest cause of stillbirths and subject to national guidelines and reporting requirements. Antenatal detection of SGA allows clinicians to undertake further investigations and plan for a timely delivery.

## Methods

The use of Power BI instead of a solution requiring software development allowed the data analysis team to be in control of creating the application and facilitate a streamlined updating process. We used the dashboard feature, dropdowns and radio buttons to display statistics relating to the rate of identification of SGA at antenatal ultrasound scan as a proportion of all babies that are SGA at birth (sensitivity) as well as false positivity. The dashboard is contained within the web-based application to monitor growth during antenatal care. Applying the row level security feature with DAX formula, we were able to personalise the report for each Trust depending on who logged into the web-app.

## Results

The application facilitates monitoring performance of the service in real time, longitudinally by month, quarter and year as well benchmark cross-sectionally against network/regional and national averages. The dashboard lets clinicians access their information in a clear and secure manner without the need for a separate link. The ready availability of data allows Trusts to enact policies to improve their performance and ultimately prevent avoidable deaths, and has contributed to the year on year decline in stillbirth rates in units that have been running this application.

## Conclusion

Development of this dashboard has resulted trusts being more aware of their own data to promote improvements in antenatal care.

